# Reflection Characteristics Measurements of Indoor Wireless Link in D-Band

**DOI:** 10.3390/s22186908

**Published:** 2022-09-13

**Authors:** Mingxu Wang, Yanyi Wang, Weiping Li, Junjie Ding, Chengzhen Bian, Xinyi Wang, Chao Wang, Chao Li, Zhimeng Zhong, Jianjun Yu

**Affiliations:** 1 Department of Communication Science and Engineering, and the Key Laboratory for Information Science of Electromagnetic Waves (MoE), Fudan University, Shanghai 200433, China; 2 Wireless Network RAN Research Department, Shanghai Huawei Technologies Co., Ltd., Shanghai 201206, China

**Keywords:** D-band, indoor wireless link, MMSE criterion, reflection characteristics, relative permittivity, surface roughness

## Abstract

For the millimeter wave (mm-Wave) and terahertz (THz) indoor wireless communication system, the reflection channels need to be characterized and modeled. In this paper, the reflection measurements of the parallel polarized wave are carried out under multiple incident angles and five kinds of materials in the D-band (110–170 GHz). A modified reflection model with two parameters estimated by the minimum mean square error (MMSE) criterion is proposed. The results show that the measurements are in good agreement with the proposed model. Furthermore, a set of measured properties is demonstrated and it can be concluded that both the reflection coefficients and relative permittivity gradually decrease, whereas the surface roughness increases slightly with the increasing frequency, indicating a weak frequency dependence. Interestingly, the concrete board with high surface roughness, which means more power loss in a specular direction, has the lowest reflection loss at a certain frequency and incident angle. It implies that the reflection characteristics of indoor building materials are determined not only by surface roughness, but also by many other factors, such as relative permittivity, frequency, and incident angle. Our work suggests that the reflection measurements of indoor D-band wireless links have a prospective application for future indoor wireless communication systems.

## 1. Introduction

By 2022, global IP traffic will reach an annual run rate of 4.8 zettabytes per year, which is 11 times more than all IP traffic generated in 2012 (437 exabytes) [1]. The huge data traffic is mainly caused by some new applications such as autonomous driving, 4 K/8 K ultra-high-definition video, centimeter-level position location, wireless backhaul and so on [2,3,4,5]. In order to meet the increasing demand of data rates, higher carrier frequency in millimeter wave (mm-Wave) range (30–300 GHz) and terahertz (THz) range (0.3–10 THz) with a much wider bandwidth has been widely studied up to now [6,7,8,9,10]. Deterministic propagation models using the ray tracing method are usually applied in mm-Wave and THz link [11,12,13]. However, due to the short wavelength of the mm-Wave and THz signals, they will suffer a great loss through obstacles. It is better to deploy an isolated indoor cellular system for communication [14,15].

Generally, the indoor environment is quite complex due to different kinds of obstacles and many corners, so there exists multipath reflection. For a better design of indoor wireless pico-cellular communication system, the characteristics of propagation channels, especially for the reflection part, need to be modeled and explored in detail [16,17]. The related work on reflection characteristics exploration is summarized in Table 1.

To establish the realistic models of indoor reflection channels, it is necessary to obtain the dielectric properties of typical building materials such as relative permittivity, surface roughness, absorption coefficient, and refractive index. Hardened concrete relative permittivity in 1.0–95.9 GHz is measured as 6.2–7.0 [24]. Other related work is summarized in Table 2. Table 3 shows the extracted permittivity of five building materials from previous research.

The studies in Table 1 provide potential solutions for indoor wireless propagation in mm-Wave and THz range. However, to the best of our knowledge, there are few studies on the reflection properties of interior building materials in the whole D-band (110–170 GHz). What’s more, the measurements of these properties in Table 2 are complex and time-consuming in reflection modeling due to the lack of devices with a high-frequency band and high resolution. Therefore, in this paper, we demonstrate the reflection measurements of the parallel polarized wave under the multiple incident angles and five kinds of materials in the whole D-band, and proposed a method based on the theoretical reflection model to estimate two dielectric properties of materials by minimum mean square error (MMSE) criterion. This method can also prove the feasibility and robustness of our experiment so that the D-band indoor reflection characteristics can be explored. The paper’s structure is as follows. The principles of reflection measurements, theoretical reflection model, and MMSE criterion are introduced in Section 2. Section 3 illustrates the procedure of our method. Section 4 presents the experimental setup of the reflection measurements and the method of data processing. In Section 5, the measured results are compared with the values theoretically calculated by the model. Additionally, in this section some characteristics, such as reflection coefficients, relative permittivity, and surface roughness of materials, are analyzed and discussed. Conclusions wrap up this paper in Section 6.

## 2. Principle of Reflection Measurements and Theoretical Model

### 2.1. Reflection Measurement

The received power of LoS transmission in free space can be obtained by the Friis transmission equation as follow:

(1) $$P_{LoS}=P_{t}\cdot {\left(\frac{\lambda }{4\pi d_{LoS}}\right)}^{2}\cdot G_{r}\cdot G_{t},$$

where $P_{t}$ is the power of transmitting antenna, $G_{t}$ and $G_{r}$ represent the gains of the transmitting and receiving antenna respectively, λ denotes wavelength, and $d_{LoS}$ defines the LoS propagation distance [32,33].

**Figure 1:**
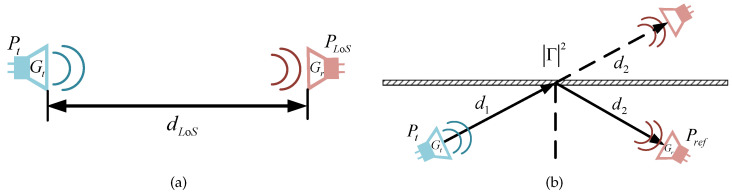
Transmission in free space. (**a**) LoS propagation measurement, (**b**) Reflection measurement.

When the electromagnetic wave hits the object at a certain angle, it usually reflects and forms a reflected wave. The ratio of the amplitudes of the reflected wave to the incident one is defined as the reflection coefficient |Γ|. The received power after reflection can be regarded as the LoS value for the unfolded path length multiplied by ${\left|\Gamma \right|}^{2}$, assuming that the size of the reflecting boundary is much larger than distances $d_{1}$ and $d_{2}$, and the surface area is much larger than the illuminated part [34], as shown in (2),

(2) $$P_{ref}=P_{t}\cdot {\left(\frac{\lambda }{4\pi \left(d_{1}+d_{2}\right)}\right)}^{2}\cdot G_{r}\cdot G_{t}\cdot {\left|\Gamma \right|}^{2},$$

where $d_{1}$, $d_{2}$ are the distances from Tx/Rx antenna to reflected object, and |Γ| refers to the reflection coefficient. Figure 1 illustrates this more intuitively. Equation (3) is obtained by dividing (2) by (1),

(3) $$\begin{array}{cc} \left|\Gamma \right| & =\frac{d_{1}+d_{2}}{d_{LoS}}\cdot \sqrt{\frac{P_{ref}}{P_{LoS}}}=\frac{d_{1}+d_{2}}{d_{LoS}}\cdot 10^{\left[P_{ref}\;\left(\mathrm{dBm}\right)-P_{LoS}\;\left(\mathrm{dBm}\right)\right]/20}, \end{array}$$

where $P_{ref}$ (dBm) and $P_{LoS}$ (dBm) are received power in reflection and LoS path in dBm, respectively. It shows that the measured reflection coefficient is determined by the difference between two kinds of received power. Additionally, the ratio of the transmission distances acts as a weighting coefficient.

### 2.2. Reflection Theoretical Model

For nonmagnetic materials, the Fresnel reflection coefficient of the parallel polarized wave is shown as (4),

(4) $$\begin{array}{cc} \left|\Gamma_{//}\right| & =\left|\frac{\frac{\varepsilon_{r2}}{\varepsilon_{r1}}\cdot \cos\theta_{i}-\sqrt{\frac{\varepsilon_{r2}}{\varepsilon_{r1}}-\sin^{2}\theta_{i}}}{\frac{\varepsilon_{r2}}{\varepsilon_{r1}}\cdot \cos\theta_{i}+\sqrt{\frac{\varepsilon_{r2}}{\varepsilon_{r1}}-\sin^{2}\theta_{i}}}\right|=\left|\frac{\varepsilon_{r}\cdot \cos\theta_{i}-\sqrt{\varepsilon_{r}-\sin^{2}\theta_{i}}}{\varepsilon_{r}\cdot \cos\theta_{i}+\sqrt{\varepsilon_{r}-\sin^{2}\theta_{i}}}\right|, \end{array}$$

where $\theta_{i}$ is the incident angle, $\epsilon_{r1}$ and $\epsilon_{r2}$ denote the relative permittivity of incident medium and reflecting medium respectively, and $\epsilon_{r}$ is the ratio of $\epsilon_{r2}$ to $\epsilon_{r1}$. Generally, the incident medium is air ($\epsilon_{r1}=1$), so $\epsilon_{r}$ is equal to $\epsilon_{r2}$.The reflection coefficient in (4) is just suitable for a completely smooth surface, because the specular reflection without scattering only occurs for the ideal surface. Actually, there is no completely smooth plane at all, so when the electromagnetic wave hits a rough surface, it will scatter at the other angle besides the reflection angle, thus causing the energy reduction in specular reflection. Therefore, (4) needs to be modified.

The first step is to characterize the roughness of the surface using Rayleigh’s rule. Rayleigh criterion defines the critical height of the surface, which is determined by the wavelength and the incident angle, as shown in (5),

(5) $$h_{c}=\frac{\lambda }{8\cos\theta_{i}}.$$

If the maximum height drop of the surface is greater than $h_{c}$ at a certain wavelength and an incident angle, the surface of the object is considered rough. On the contrary, the surface of the object is smooth [35]. The effects of the scattering can be modeled by plenty of simulations based on the Maxwell boundary value problem [36]. Instead, there has been a simpler analytic approximation method applied to D-band scattering issue [20]. The previous descriptions have shown that the surface roughness is usually characterized by two parameters, namely root mean square height and correlation length [37]. Here, the root mean square height is defined as the standard deviation of the surface height from the average height, which is used to characterize the surface roughness in this paper, and the larger it is, the rougher the surface will be.

In order to mitigate the energy loss caused by the scattering in specular reflection, the Rayleigh roughness factor from Beckman-Kirchhoff theory [38] is introduced, as illustrated in (6) [35,39],

(6) $$\rho_{s}=\exp\left[-8\cdot {\left(\frac{\pi h_{rms}\cos\theta_{i}}{\lambda }\right)}^{2}\right]\cdot J_{0}\left[8\cdot \left(\frac{\pi h_{rms}\cos\theta_{i}}{\lambda }\right)\right],$$

where λ is the wavelength, $\theta_{i}$ is the incident angle, $h_{rms}$ denotes the root mean square height, namely surface roughness, and $J_{0}\left(\cdot \right)$ is the first type of zero-order Bessel function. Gaussian height distribution of the surface is assumed, and the sharp edge and shadowing can be neglected in this model. Therefore, the modified reflection coefficient is as follows:

(7) $$\begin{array}{cc} \left|\Gamma_{//}\right| & =\rho_{s}\cdot \left|\frac{\varepsilon_{r}\cdot \cos\theta_{i}-\sqrt{\varepsilon_{r}-\sin^{2}\theta_{i}}}{\varepsilon_{r}\cdot \cos\theta_{i}+\sqrt{\varepsilon_{r}-\sin^{2}\theta_{i}}}\right|. \end{array}$$

### 2.3. Minimum Mean Square Error Criterion

Mean square error (MSE) represents the matching degree between the predicted values and the real values, and it usually acts as the loss function of regression problems, as shown in (8),

(8) $$MSE=\frac{1}{n}\sum_{i=1}^{n}{\left(Y_{i}-{\overset{\wedge }{Y}}_{i}\right)}^{2},$$

where ${\overset{\wedge }{Y}}_{i}$ is the predicted values, $Y_{i}$ is the real values, and n is the number of samples. When the model has been established, whereas some parameters are not determined, these unknown parameters can be estimated by taking the minimum MSE, which is called the MMSE criterion. In this paper, the theoretical model has been built, so the MMSE criterion will play an important role in parameters fitting and results verification.

## 3. Methodology

As introduced in Section 2, the reflection coefficient can be calculated by the theoretical model. However, (6) and (7) indicate that if the parallel polarization reflection coefficient is to be calculated through the theoretical model, it is necessary to determine the value of λ, $\theta_{i}$, $h_{rms}$ and $\epsilon_{r}$. In fact, λ, and $\theta_{i}$ are easy to measure, whereas $h_{rms}$ and $\epsilon_{r}$ are difficult due to the requirement of the sophisticated devices. In order to reduce the cost and improve the efficiency of the experiment, we propose a method employing the MMSE criterion to simultaneously estimate the relative permittivity and surface roughness, and also optimize them. The estimated parameters are further compared with the data in previous research.

The proposed method is introduced in detail as follows:

1. The reflection coefficients, expressed as ${\left|\Gamma \right|}_{M}$, are measured based on Section 2.1;
2. The ranges of two parameters, $\epsilon_{r}$ and $h_{rms}$, are set to calculate the theoretical reflection coefficients ${\left|\Gamma \right|}_{T}$;
3. The dataset of theoretical ${\left|\Gamma \right|}_{T}$ is compared with that of measured ${\left|\Gamma \right|}_{M}$ to calculate the MSE;.
4. With MMSE criterion, the set of theoretical ${\left|\Gamma \right|}_{T}$ closest to the measured ${\left|\Gamma \right|}_{M}$ is selected, and therefore, the optimal relative permittivity $\epsilon_{r}$ and optimal surface roughness $h_{rms}$ are obtained in two ranges;
5. These two optimal estimated parameters are compared with the data in previous research to prove the rationality and robustness of our method and experiment;
6. Based on the steps above, the reflection characteristics can be analyzed.

**Figure 2:**
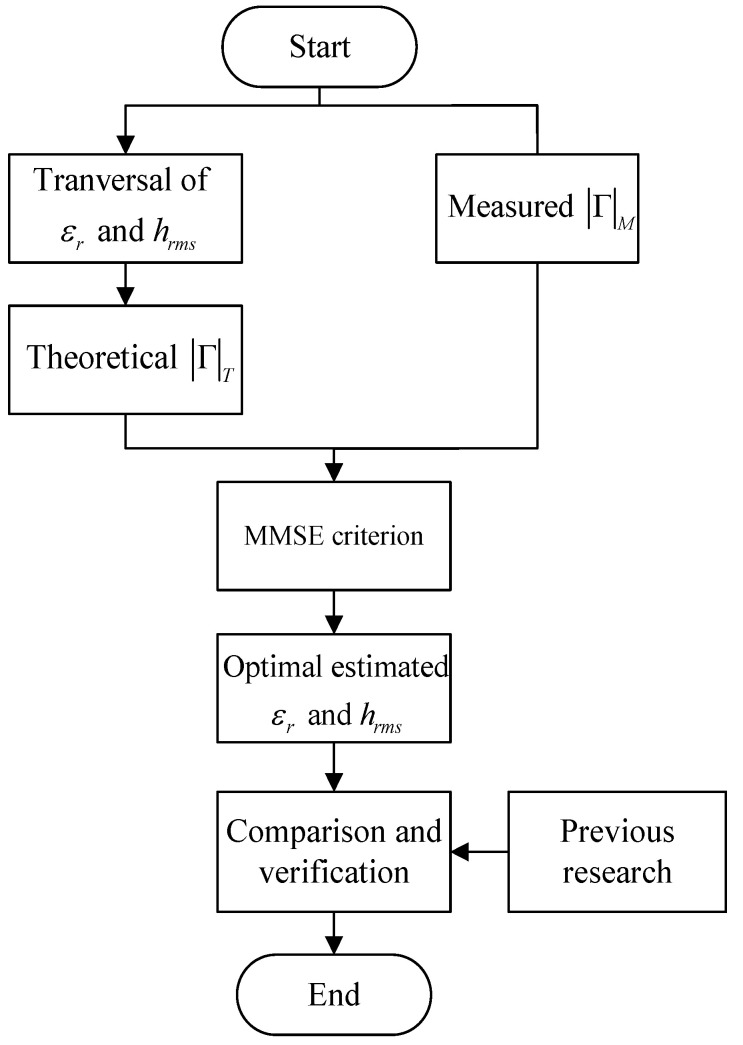
The flow chart of dataset processing.

This process is depicted in Figure 2.

## 4. Experimental Setup

**Figure 3:**
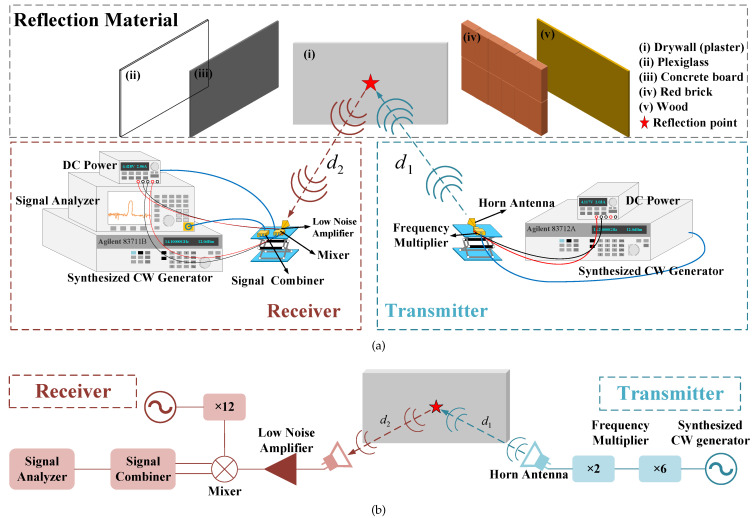
Transmission in free space. (**a**) LoS propagation measurement, (**b**) Reflection measurement.

Figure 3 demonstrates the experimental setup of the reflection measurement in the D-band indoor wireless link. The narrowband signal in D-band is generated from the synthesized continuous wave generator. After up-conversion via two cascaded frequency multipliers (i.e., ×2 and ×6 respectively), the narrowband D-band signal is transmitted to the free space by the standard horn antenna with a gain of 25 dBi, and then reflected by different types of materials. The reflected signal is received by the other standard horn antenna (HA), which is the same as the HA at the transmitter side.

In the receiving end, the received signal is amplified by a low noise amplifier (LNA) with a gain of 35 dB, and then down-converted in a mixer. The local oscillator (LO) signal is generated by a continuous wave generator and up-converted by a twelve times frequency multiplier. After down-conversion, the intermediate frequency (IF) signal passes through a signal combiner and is finally measured by a signal analyzer to show its spectrum. Therefore, the characteristics of the reflected signal including frequency and power can be obtained. In order to analyze the reflection characteristics in the full D-band range, seven D-band signals with a frequency interval of 10 GHz are generated for the measurements. The detailed hardware parameters are demonstrated in Table 4.

**Figure 4:**
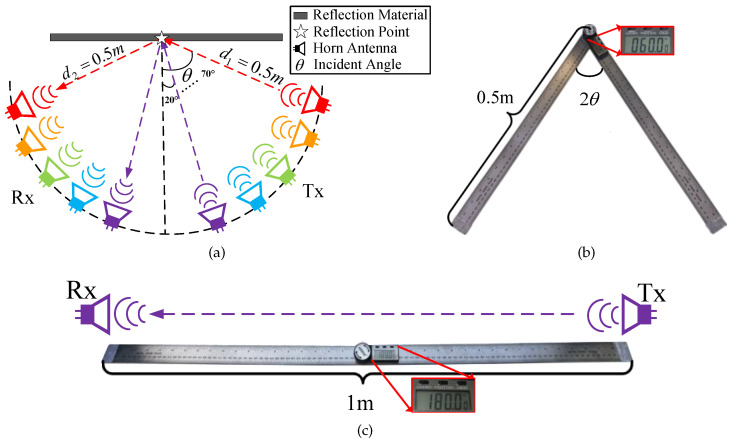
(**a**) Design of reflection measurements, (**b**) Angle ruler, (**c**) Design of LoS propagation.

**Figure 5:**
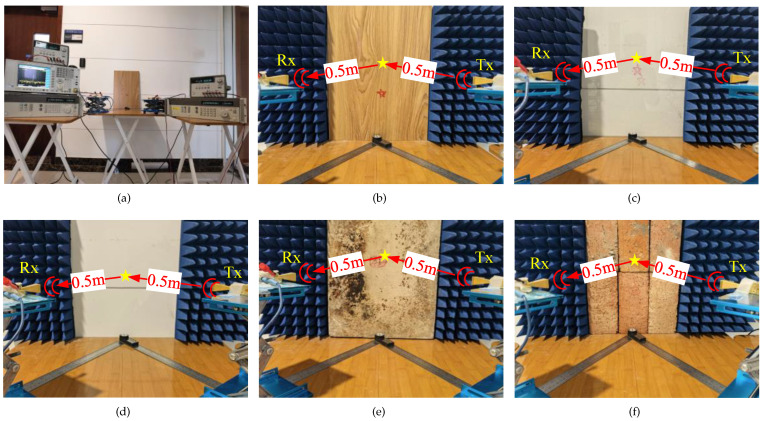
(**a**) The indoor reflection measurement campaigns in D-band for five materials, (**b**) Wood, (**c**) Plexiglass, (**d**) Drywall (plaster), (**e**) Concrete board, (**f**) Red brick.

For the reflection loss measurements, in order to ensure that Tx and Rx antennas are located in the far field region of the antennas, the distances to the reflective materials should be greater than Fraunhofer distance, i.e.,

(9) $$d>\frac{2D^{2}}{\lambda },$$

where *D* represents the horn antenna aperture size [40]. According to (8), the maximum Fraunhofer distance in this system is about 0.27 m, so the distances $d_{1}$ and $d_{2}$ are both set as 0.5 m. This distance is a compromise between far-field conditions and illumination of the reflective materials by the antennas [41]. Additionally, (4) indicates that the incident angle has an influence on the reflection coefficient and further affects the received power. Therefore, for the narrowband signals at different frequencies, six angles changing from 20° to 70° are selected as incident angles for measurements, as shown in Figure 4a [40]. Both the angles and distances are measured by a high precision angle ruler in Figure 4b to minimize the calibration error. In fact, different materials have different properties. In order to explore the reflection characteristics of an indoor wireless link in D-band, five kinds of materials, i.e., wood, plexiglass, drywall (plaster), concrete board, and red brick, are selected as the reflection media. The measurement scenarios are shown in Figure 5.

Table 5 demonstrates the sizes of materials and they are much larger than the first Fresnel zone radii in D-band. Based on the experiments above, the dataset of the received power in reflection measurements can be collected. According to (3), in order to calculate the reflection coefficients, the received power in LoS propagation is also tested under the same experimental conditions as reflection measurements, including transmitted power, distance, frequency, experimental environment, etc., as demonstrated in Figure 4c. Finally, the dataset of the average received power of five measurements in reflection and LoS paths are obtained to calculate the reflection coefficients by (3). As mentioned in Section 3, with these measured reflection coefficients, the optimal relative permittivity and surface roughness of different materials can be estimated, and then the reflection characteristics will be discussed.

## 5. Results and Discussions

**Figure 6:**
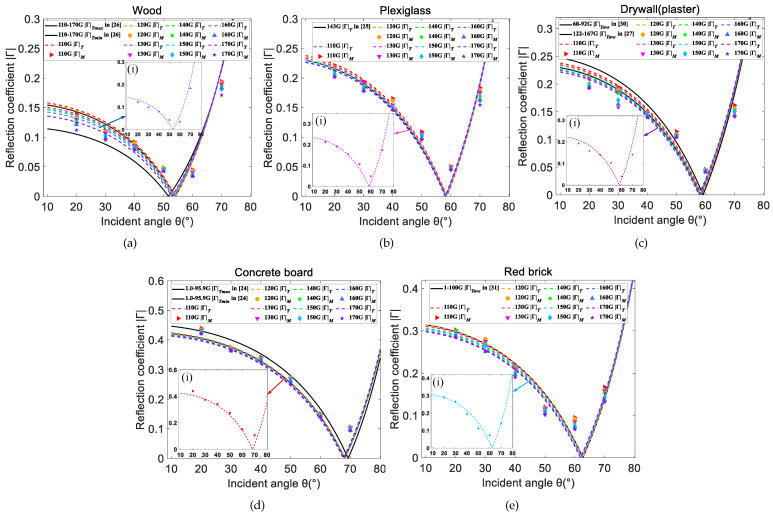
Comparison between measured and Fresnel reflection coefficients for five materials [24,25,26,27,30,31]. (**a**) Wood, (**b**) Plexiglass, (**c**) Drywall (plaster), (**d**) Concrete board, (**e**) Red brick.

Figure 6a–e show the measured reflection coefficients of five kinds of materials in the D-band range, compared with the theoretical values using modified Fresnel reflection Formula (7). These symbols represent the measured reflection coefficients, which are calculated by the reflection loss [$P_{ref}\;\left(\mathrm{dBm}\right)$−$P_{LoS}\;\left(\mathrm{dBm}\right)$] in Table 6 using (3), whereas the group of broken lines denotes the theoretical results. What’s more, the black solid lines are plotted on the basis of (7) and previous research about relative permittivity shown in Table 3. They act as reference lines for more intuitive comparison and analysis. ${\left|\Gamma \right|}_{T}$ in legends represents the theoretical reflection coefficient and ${\left|\Gamma \right|}_{M}$ denotes the measured value. The optimal estimated relative permittivity $\epsilon_{r_{E}}$ and surface roughness $h_{rms}$ of the materials in the full D-band range are summarized in Table 7. $\epsilon_{r_{R}}$ represents the results of previous research. MMSE${}_{ave}$ is the average minimum mean square error between the measured ${\left|\Gamma \right|}_{M}$ and theoretical ${\left|\Gamma \right|}_{T}$ from 110 GHz to 170 GHz.

### 5.1. Reflection Coefficients

As can be seen from Figure 6a–e, the measured reflection coefficients fit well with the theoretical model and they are comparable in spite of the small deviation. The MMSE, which are on the order of −4 in Table 7, also back this up. The plexiglass has the lowest MMSE among the five materials which means a better consistency between ${\left|\Gamma \right|}_{M}$ and ${\left|\Gamma \right|}_{T}$, whereas the concrete with the highest MMSE shows a worse consistency. Both the measured and theoretical reflection coefficients are a decreasing function for the angles smaller than the Brewster angle, whereas they increase rapidly with angles larger than the Brewster angle. When the angle rises up to 90°, the reflection coefficient will be 1 because the main transmission path is not a reflection but an LoS path. This conforms to the properties of parallel polarized wave [17,20]. The trend is more clear in insets (i) of Figure 6a–e, which demonstrate the specific fitting situations. The Brewster angles of different materials at a certain frequency vary greatly. The wood’s Brewster angle is close to 50°, while that of the concrete board is almost 70°. The remaining three materials have Brewster angles of about 60°. The difference is caused by the dielectric properties of materials. Additionally, at a certain angle smaller than the Brewster angle, the theoretical reflection coefficients decrease slightly from 110 GHz to 170 GHz, but some measured results don’t follow it. For example, the measured reflection coefficient of plexiglass at 120 GHz is smaller than that at 130 GHz and the measured reflection coefficient of red brick at 170 GHz is larger than that at 160 GHz. This is attributed to the limited resolution of devices and environmental interference.

**Figure 7:**
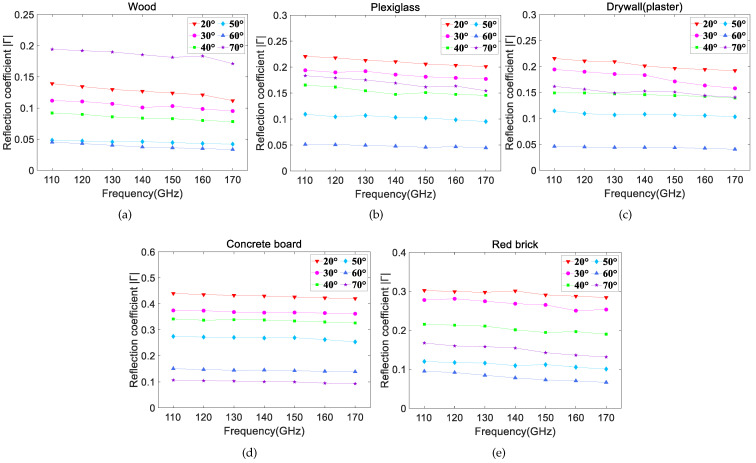
The relationship between measured reflection coefficients of five materials and frequencies in D-band. (**a**) Wood, (**b**) Plexiglass, (**c**) Drywall (plaster), (**d**) Concrete board, (**e**) Red brick.

Figure 7a–e shows the relationship between the measured reflection coefficients and the frequencies at different incident angles. It can be seen that the reflection coefficients demonstrate an overall downward trend with slight fluctuations from 110 GHz to 170 GHz at a certain angle, and the trend is flat. This is expected, because with increasing frequency, the Rayleigh roughness factor (described by $\rho_{s}$) of the material grows and the energy in the specular direction decreases, leading to the reduction in the reflection coefficient [20]. At a certain frequency, the reflection coefficients decrease with the incident angle (i.e., 20°, 30°, 40°, 50°, 60°). Additionally, the measured reflection coefficients are generally small and most of them are less than 0.3, implying a high reflection loss.

### 5.2. Relative Permittivity

Through the analysis of the monotonicity between the $|\Gamma_{//}|$ and $\epsilon_{r}$ in (7), it can be concluded that the Fresnel reflection coefficients will improve as the relative permittivity increases. The rank of the broken lines with different relative permittivity in Figure 6a–e also verifies this conclusion. In addition, by comparing the estimated relative permittivity with the published data in Table 7, it can be concluded that the estimated dielectric constants of materials in the D-band are still relatively accurate with a slight deviation. It can be also found that the broken lines in Figure 6a–e are basically distributed around the black solid lines, although the test frequencies of relative permittivity corresponding to the black lines in Figure 6d,e do not reach the D-band range [24,42]. This is attributed to the weak frequency dependence of the dielectric constant. The small deviation between the estimated relative permittivity and the previously published results is caused by the following reasons [27]. First, there are differences in the materials, i.e., the exact composition and surface roughness. Second, no measurement can eliminate the error completely, which will lead to discrepancies. Last but not least, different processing methods of the dataset and different experimental environments also play a role. Overall, the differences are acceptable, and our results are comparable to those in previous references. This indicates that the measurement campaigns, the established model, and dataset processing proposed in this paper are reliable and robust.

**Figure 8:**
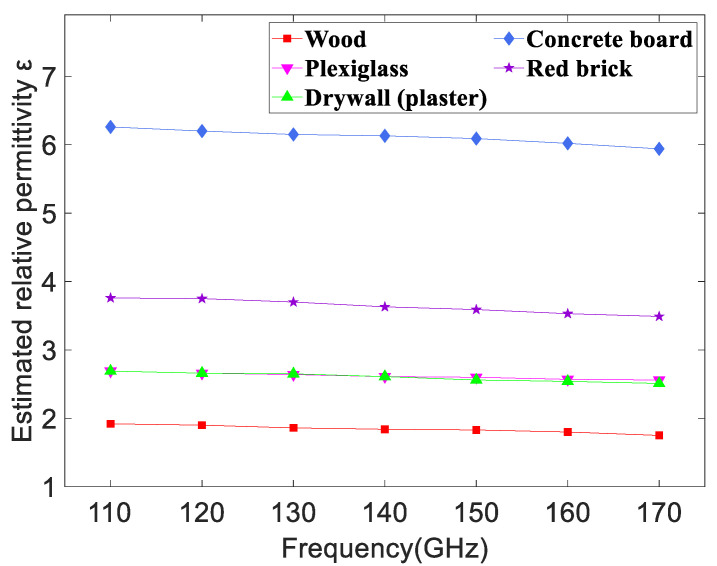
The relationship between estimated relative permittivity of five materials and frequencies in D-band.

The previous research suggests that the relative permittivity of the samples does not change significantly with the transmission frequency, even over a wide frequency range. It is also found that the dielectric constant is usually related to the variation of sample compositions rather than the frequency [43,44]. Figure 8 demonstrates the estimated relative permittivity of five kinds of materials versus the frequency. It is easy to conclude that the relative permittivity decreases with slight fluctuations from 110 GHz to 170 GHz for a certain material, but the change is quite subtle. For example, the relative permittivity of wood decreases from 1.92 to 1.75 with increasing frequency in D-band and the largest gap is only 0.17, which is less than one-tenth of the relative permittivity. In the same way, the largest gaps are just 0.13, 0.18, 0.32, and 0.27 respectively, corresponding to plexiglass, drywall (plaster), concrete board, and red brick. In addition, the relative permittivity of different materials changes significantly at a certain frequency. The values of concrete board and wood are the largest and smallest, respectively. The results in Figure 8 are consistent with the previous research and further confirm the reliability of our work.

### 5.3. Surface Roughness

**Figure 9:**
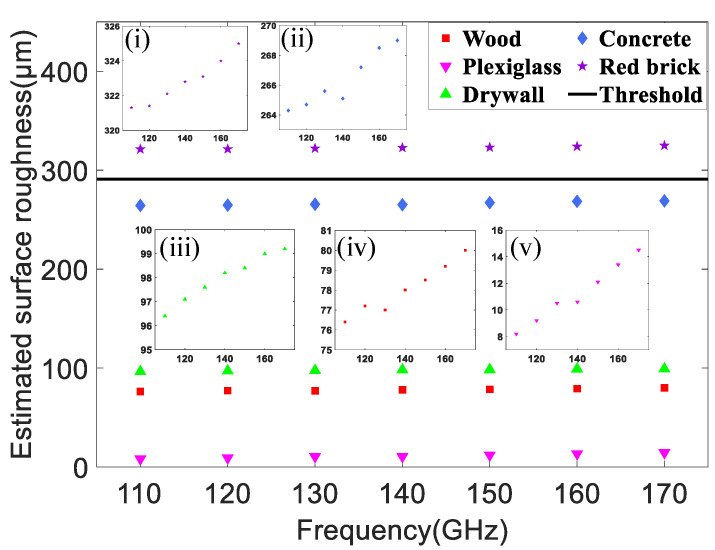
The relationship between estimated surface roughness of five materials and frequencies in D-band.

Figure 9 demonstrates the surface roughness of five kinds of materials in the D-band range. The symbols denote the estimated surface roughness by the MMSE criterion and the average of them are 78.0 µm, 11.2 µm, 97.9 µm, 266.7 µm, and 322.8 µm respectively, corresponding to wood, plexiglass, drywall (plaster), concrete board and red brick. It is evident from the insets (i)–(v) of Figure 9 that the surface roughness increases with frequency but the change is small. The reason is that the signal at higher frequency suffers a larger penetration loss. Therefore, the signal distribution will be concentrated on the surface and more scattering will occur, which increases the roughness of surface [31]. The black bold line is set as the threshold (291 µm) to distinguish whether the surface of an object is rough or smooth. It is determined by (5) and [45]. Therefore, the surface of plexiglass is regarded as quite smooth. The wood and drywall (plaster) are considered relatively smooth although they are rougher than the plexiglass. Note that the surface roughness of the concrete board is so close to the threshold that it is classified as rough material like red brick. As can be seen from Table 7, the smooth plexiglass has the lowest MMSE whereas the rough materials concrete and red brick have higher values. Chances are that the MMSE can imply the surface roughness of building materials in our method. Despite the high surface roughness of the concrete board, which means more power loss in the specular direction, its reflection loss is lower than that of the other four kinds of materials shown in Table 6. In fact, based on the analysis of (7) before, it is not surprising that the concrete board has the lowest reflection loss of the five kinds of materials because it has the largest relative permittivity. This suggests that the reflection characteristics of indoor building materials are determined not only by surface roughness, but also by many other factors, such as relative permittivity, frequency, and incident angle.

## 6. Conclusions

For five kinds of materials, we present a series of indoor reflection measurements at six incident angles (i.e., 20°, 30°, 40°, 50°, 60°, and 70°) in the full D-band range. We also establish a modified theoretical reflection model with two parameters (i.e., relative permittivity and surface roughness) fitting by the MMSE criterion. With this model, it is unnecessary to measure these two parameters by complex devices because they can be estimated by this model. The estimated relative permittivity from 110 GHz to 170 GHz is 1.75–1.92, 2.56–2.69, 2.51–2.69, 5.94–6.26, 3.49–3.76, respectively and the estimated surface roughness is 76.4–80.0 µm, 8.2–14.5 µm, 96.4–99.2 µm, 264.3–269.0 µm and 321.3–325 µm respectively, corresponding to wood, plexiglass, drywall (plaster), concrete board and red brick. These estimated parameters of materials are very close to the data in previous references and the measured reflection coefficients are in good agreement with the theoretical values calculated by the model. Through the analysis of the relationships between properties and frequency in the D-band, it is concluded that both the reflection coefficients and the relative permittivity demonstrate a slow downward trend, whereas the surface roughness increases slightly with increasing frequency. They all demonstrate a weak frequency dependence. The large reflection loss suggests that the parallel polarized wave may be not suitable for indoor wireless communication in D-band. Interestingly, at a fixed angle and frequency, the concrete board with the highest roughness have the lowest reflection loss, indicating that the reflection characteristics of indoor building materials are determined not only by surface roughness, but also by many other factors, such as relative permittivity, frequency, and incident angle. Although our results are obtained in the full D-band range, we believe that the general conclusions remain applicable even for higher frequency in the THz range. Our work suggests many optimistic possibilities for an indoor wireless pico-cellular communication system. In the future, we want to study the indoor channel transmission characteristics in a larger space, including multiple reflections and multipath delay, and explore the transmission performance of data flow in THz indoor channels. 

## Figures and Tables

**Table 1:** Reference Review of Exploring Reflection Characteristics.

Year	Frequency	Contributions
1995	57.5, 78.5 and 95.9 GHz	The authors put forward layer models for estimating the refractive indices; The authors used a network-analyzer-based step-frequency radar system to measure the reflection coefficients of multiple construction materials [18].
1997	60 GHz band	The authors measured the reflection characteristics of some typical materials; The authors confirmed that the circular polarization wave will reduce the reflection energy compared with linear polarization even though the building materials have complicated structures [19].
2007	100–1000 GHz band	The authors introduced a Rayleigh roughness factor calculated from the measured surface height distribution of the sample and derived modified Fresnel equations using Kirchhoff scattering theory; The authors derived reflection coefficients based on material parameters and surface measurements in propagation models and compared them with the measured results. Both of them shows good agreement [20].
2008	100–500 GHz	The authors provided measurements and modeling results of multiple reflections in building materials; In contrast to bulk materials, the reflection losses show strong oscillations over the frequency which result from interference [21].
2010	110–135 GHz	The authors studied the measured and simulated wideband reflection properties of different objects and showed good agreement [22].
2018	100, 200, 300 and 400 GHz	The authors characterized THz wireless links using a 1 Gbit/s data flow in both indoor and outdoor environments; The authors establish the feasibility of using THz carrier waves for data transmission [23].

**Table 2:** Reference Review of Measuring Dielectric Properties of Materials.

Year	Materials	Dielectric Properties	Equipment	Frequency	Results
1966	Plexiglass	Permittivity	Fabry-Perot resonator	143 GHz	2.60 [25]
2005	Plaster, glass and wood	Absorption coefficient and refractive index	THz time-domain spectroscopy	70–350 GHz	Figures to show the measured parameters VS frequency [17].
2007	Ingrain wallpaper and two plaster samples	Surface roughness	Commercially available equipment for optical 3D micro- and nanometrology	100–1000 GHz	σ of the surface height is 0.13 mm, 0.05 mm and 0.15 mm, respectively [20].
2014	Four wood species	Complex permittivity	Quasioptical Mach-Zahnder Interferometer with backward-wave oscillator	100–500 GHz	There is slight deviation among species, but the overall range is 1.60–1.89 [26].
2019	Gypsum	Permittivity	Frequency-modulated continuous-wave radar sensors	122–169 GHz	2.595 [27]

**Table 3:** Permittivity Review of Indoor Materials.

Material	Frequency	Permittivity
Wood	1–100 GHz	1.99 [28]
	110–170 GHz	1.60–1.89 [26]
Plexiglass	143 GHz	2.60 [25]
	60–300 GHz	2.581–2.602 [29]
Drywall (plaster)	68–92 GHz	2.80 [30]
	122–167 GHz	2.595–2.602 [27]
Concrete board	1–95.9 GHz	6.2–7.0 [24]
Red brick	1–100 GHz	3.75 [31]

**Table 4:** D-band Reflection System Specifications.

Specifications	Values						
Center frequency (GHz)	110	120	130	140	150	160	170
LO frequency (GHz)	9.06	9.90	10.73	11.56	12.40	13.23	14.06
IF frequency (GHz)	1.2						
Tx/Rx antenna gain (dBi)	25						
Tx/Rx azimuth HPBW	E plane: 9°/H plane: 10°						
Tx/Rx polarization	Horizontal						
Tx/Rx caliber (mm × mm)	17.5 × 13.6						
Tx/Rx projection diameter (mm)	19.1						
LNA gain (dB)	35						

**Table 5:** Size of Materials and First Fresnel Zone.

Material	Size (cm × cm)	Radius of First Fresnel Zone in D-Band (cm)
Wood	49.2 × 35.8	2.10–2.61
Plexiglass	88.2 × 42.9	
Drywall (plaster)	56.1 × 37.5	
Concrete board	59.7 × 39.3	
Red brick	45.6 × 31.2	

**Table 6:** The Average Reflection Loss [$P_{ref}\;\left(\mathrm{dBm}\right)$−$P_{LoS}\;\left(\mathrm{dBm}\right)$] at Six Incident Angles in D-band.

Material	Reflection Loss (dB)					
	20°	30°	40°	50°	60°	70°
Wood	17.3	19.1	20.8	26.3	27.7	14.0
Plexiglass	12.9	14.0	15.7	19.1	25.8	14.8
Drywall	13.2	14.4	16.1	18.7	26.6	15.8
Concrete	6.7	8.1	8.9	10.8	16.2	19.3
Red brick	10.0	10.8	13.2	18.4	21.4	15.9

**Table 7:** Collection of Estimated Parameters and MSE.

Material	$\epsilon_{r_{R}}$	$\epsilon_{r_{E}}$	$h_{\mathrm{rms}}$ (µm)	MMSE${}_{ave}$
Wood	1.60–1.89	1.75–1.92	76.4–80.0	3.79 × $e^{-4}$
Plexiglass	2.581–2.602	2.56–2.69	8.2–14.5	3.04 × $e^{-4}$
Drywall	2.595–2.602	2.51–2.69	96.4–99.2	5.47 × $e^{-4}$
Concrete	6.2–7.0	5.94–6.26	264.3–269.0	7.25 × $e^{-4}$
Red brick	3.75	3.49–3.76	321.3–325.0	6.43 × $e^{-4}$

## Data Availability

Not applicable.
